# Interdisciplinary insights into the link between gut microbiome and gastric carcinogenesis—what is currently known?

**DOI:** 10.1007/s10120-021-01260-y

**Published:** 2021-11-06

**Authors:** Karolina Kaźmierczak-Siedlecka, Agnieszka Daca, Giandomenico Roviello, Martina Catalano, Karol Połom

**Affiliations:** 1 Department of Surgical Oncology, Medical University of Gdansk, ul. Smoluchowskiego 17, 80-214 Gdańsk, Poland; 2 Department of Pathology and Experimental Rheumatology, Medical University of Gdansk, Gdańsk, Poland; 3 Department of Health Sciences, Section of Clinical Pharmacology and Oncology, University of Florence, Viale Pieraccini, 6, 50139 Florence, Italy

**Keywords:** Gastric carcinogenesis, Gastric microbiome, Mycobiota, Microbial metabolites

## Abstract

Currently, gastric cancer is one of the leading death-related cancer globally. The etiopathogenesis of gastric cancer is multifactorial and includes among others dysbiotic alterations of gastric microbiota. Molecular techniques revealed that stomach is not a sterile organ and it is resides with ecosystem of microbes. Due to the fact that the role of *Helicobacter pylori* infection in development of gastric cancer is established and well-studied, this paper is mainly focused on the role of other bacterial as well as viral and fungal gut microbiota imbalance in gastric carcinogenesis. Notably, not only the composition of gastric microbiota may play an important role in development of gastric cancer, but also its activity. Microbial metabolites, such as short-chain fatty acids, polyamines, N-nitroso compounds, and lactate, may significantly affect gastric carcinogenesis. Therefore, this paper discussed aforementioned aspects with the interdisciplinary insights (regarding also immunological point of view) into the association between gut microbiome and gastric carcinogenesis based on up-to-date studies.

## Introduction

According to the report based on American Cancer Society and World Health Organization (WHO) databases, gastric cancer (GC), besides lung and liver cancer, is one of the most deadly cancers in the general population [1]. Adenocarcinoma accounts for approximately 90% of gastric cancer cases histologically divided into two major types, i.e., diffuse and intestinal according to the Lauren’s classification [2]. WHO classification recognizes four major histologic patterns of gastric cancer: papillary, tubular, mucinous and poorly cohesive (including signet ring cell carcinoma), plus uncommon histologic variants [3].

The main risk factors for gastric cancer are *Helicobacter pylori* infection, age, male gender, tobacco smoking, race, pharmacological treatment, radiation, low level of physical activity, eating habits (e.g., high consumption of smoked, salty foods and low intake of dietary fiber, vegetables, fruits), iron deficiency, obesity (especially body mass index > 40 kg/m^2^), and genetic background [2, 4, 5]. Polymorphism in pro-inflammatory IL-1β gene cluster, mainly *IL-1β-31* and *IL-1β-511,* was considered good candidate, although the innovative GWAS (genome wide association studies) did not prove these polymorphisms involvement in GC development [2, 5]. GWAS pinpointed towards other possible candidates such as SNPs (single nucleotide polymorphisms) in prostate stem cell antigen (*PSCA*) gene and gene encoding mucin 1–*MUC1* gene. Both of them are implicated in higher risk of diffuse type of GC [2], although it should be considered that, the data come mainly from East-Asia region [2].

Age affects the occurrence of gastric cancer with higher incidence in elderly population [2]. Howlader et al. reported that 29% of GC cases were patients at age between 75 and 84 years and only 1% of patients at age of 20–34 years [6]. Moreover, in Kaźmierczak-Siedlecka et al. retrospective analysis, it was noted that range of age of patients with gastric/esophageal cancer (who were qualified for home enteral nutrition) was 48–93 years whereas average age was 68 ± 10.1 years [7]. Other potential risk factors which might contribute to development of GC are poor oral hygiene, loss of tooth and use of opium [2, 8].

The etiopathogenesis of GC is multifactorial though chronic inflammation with *H. pylori* is the major risk factor. Notwithstanding, it is estimated that only 3% of people who are infected with this bacterium will develop GC eventually [9, 10] and, in some cases the tumour progression is observed even after eradication of *H. pylori* [11]. Other dysbiotic alterations of gastric microbiota might also be associated with development of GC; however, they are not well established yet. According to the data, some bacteria, fungi, and viruses which might be involved in gastric carcinogenesis exist [9]. In addition, several gut microbial metabolites affect tumorigenic pathways both positively as well as negatively. Therefore, in this review, we briefly presented gastric microbiota in healthy individuals. Then, we discussed gut microbiota imbalance (regarding bacteria, fungi, and viruses) in gastric cancer. Since most of papers described the role of *H. pylori* in gastric carcinogenesis very well, in this manuscript we mainly focused on other microorganisms than *H. pylori* carcinogenesis context. Finally, we showed the role of gut microbial metabolites in gastric carcinogenesis.

## Gastric microbiota in healthy individuals

Historically, stomach in healthy people was considered as a sterile organ [11, 12]. Acidic condition of stomach was described as unfavourable environment for colonisation with both bacteria and fungi [13]. Nowadays, molecular techniques revealed that stomach is colonised with ecosystem of microbes and that the composition of gastric microbiota varies individually and depends on several factors, such as diet, administration of antibiotics and proton pump inhibitors [14–16]. Microbes residing stomach can survive in acidic pH. Overall, *Proteobacteria*, *Firmicutes*, *Bacteroidetes*, *Fusobacteria*, and *Actinobacteria* phyla are the main bacterial components of gastric microbiota in healthy subjects [17–19]. Notably, *Lactobacillus*, *Streptococcus*, and *Propionibacterium* are the most characteristic bacterial genera which can be found in stomach [20].

The balance and relationship between gastric microbiota and host’s body are maintained by the innate lymphoid cells (ILCs), the elements of which generally are considered responsible for keeping tissue homeostasis [21]. In the stomach milieu especially the ILCs subtype called group 2 (ILC2s) seems to be important [19, 22]. While the other types of ILCs are abundant in other parts of gastrointestinal tract, ILC2s are dominant in the stomach. What is interesting, the population itself exists only in the presence of microbiota. The data from experiments on germ-free mice show evidently lower number of the ILC2s in the stomach and experiments on specific pathogen free mice strongly suggest that *Bacteroidales* order is to be responsible for the ILC2s stimulation through the induction of specific interleukins production [21, 22]. ILC2s stimulate IgA antibodies production by plasma cells and thus help in keeping balance and protecting epithelium from bacteria-mediated damage and help to eliminate IgA-coated bacteria from the body [19, 21, 22].

Fungi constitute approximately 0.2% of microorganisms in human body, thus they are integral part of microbiota [23]. The most common genera which reside human gut are *Candida*, *Saccharomyces* as well as *Cladosporium* [23, 24]*.* Intestinal fungal community play a significant role in human body. Among others, it maintains gut homeostasis, interacts with other gut microbes, and affects immune system. According to some studies, fungi are isolated from stomach of individuals without symptoms of fungal infections in wide range, i.e., 7–33% cases [25–27]. Notwithstanding, data regarding the composition of fungal microbiota in stomach are incomplete. The stability of stomach mycobiota is also not well investigated yet [28].

As our knowledge about gastric mycobiota is rather limited, the understanding of host’s immune system–fungi interaction in health is even more elusive. Some recent data coming from experiments on mice, delivered by Zhu et al. suggest that fungi can have a certain impact on tumorigenesis and cannot be ignored as potential causative agents [29]. What is also worth to remember, if certain imbalance is implicated in the development of cancer, restoring the balance may be a useful preventive and therapeutic strategy. That is a reason, why probably in a near future we will observe an increased interest in host’s immune system–gastric and gut mycobiota interactions research.

## Gut microbiota imbalance in gastric cancer

### Bacteria

*H. pylori*—class I carcinogen for gastric cancer—triggers inflammation of gastric mucosa, causes destruction of hydrochloric acid-secreting gastric glands and mucosal atrophy leading to development of gastric cancer [14, 30–32]. *H. pylori* interacts with other gastrointestinal microbes negatively correlating with the alpha diversity of gastric microbiota; *H. pylori* eradication may increase the diversity of stomach microbiota [32]. There are other bacteria which are enriched in GC and potentially they might be involved in gastric carcinogenesis [30]. *H. pylori* initiates gastric inflammation; however, other microbes might maintain and progress inflammation, dysplastic alterations and then they might cause development of GC [33]. Notably, gastric microbiota imbalance observed as a reduction of microbial diversity may cause inflammation and induce genotoxicity, thus it promotes gastric cancer development [34].

Sung et al. identified microbes which are associated with gastric inflammation, atrophy as well as intestinal metaplasia after 1 year eradication of *H. pylori* [33]*.* This study regard 587 patients (*H. pylori*-positive) which were divided into two groups: first receiving *H. pylori* eradication treatment, i.e., omeprazole (20 mg), amoxicillin (1 g) and clarithromycin (500 mg) twice daily per 1 week (*n* = 295) and second group consuming placebo (*n* = 292). Bacterial taxonomy was assessed from stomach specimens (gastric biopsy samples) using 16S rRNA sequencing method at baseline and after 1 year. The level of microbes, such as *Acinetobacter lwoffii*, *Streptococcus anginosus*, and *Ralstonia,* was increased whereas the amount of *Roseburia* and *Sphingomonas* was decreased in case of persistent inflammation after 1 year from eradication of *H. pylori*. In addition, oral microbes, such as *Peptostreptococcus*, *Streptococcus*, *Parvimonas*, *Prevotella*, *Rothia*, and *Granulicatella,* were related to persistence of atrophy and intestinal metaplasia whereas *Faecalibacterium prausnitzii* was reduced in patients who developed atrophy after 1 year of *H. pylori* eradication. The identification of aforementioned microbes in this context open a new promising therapeutic strategies for prevention of GC [33]. Recently, in another study it was shown that gastric dysbiosis may persist long period after eradication of *H. pylori* [35]. In addition, gastric microbiota imbalance may be associated with development of primary and metachronous GC after *H. pylori* eradication [35].

Several studies revealed the differences in gastric microbiota between patients with gastric cancer and control subjects [36–38]. It should be emphasized that differences in sample types, sequencing methods, geographic origin as well as environmental exposures of the population should be taken into consideration during data analysing [34]. These aspects are extremely significant during establishing microbial biomarkers, because they are multifactorial-dependent and should be validated in wide range of population. Wang et al. characterized the composition of gastric microbiota using gastric biopsies from antrum or within 5 cm of cancerous lesion [17]. This study included 315 patients (*n* = 212 chronic gastritis—controls, *n* = 103 gastric cancer; China). It was observed that gastric mucosa consists of average 6.9 × 10^8^ bacteria/gram of tissue. Five genera, such as *Lactobacillus*, *Lachnospiraceae*, *Escherichia-Shigella*, *Nitrospirae*, and *Burkholderia fungorum,* were enriched in gastric mucosa specimens in patients with GC. These microbes might be involved in carcinogenesis via several mechanisms. For instance, *Nitrospirae*, *Lactobacillus*, and *E. coli* participate in nitrate/nitrite metabolisms. N-nitroso compounds are assessed as carcinogen and they are derived from metabolism of nitrate/nitrite [17]. In another study gastric microbiota was assessed using shotgun metagenomic sequencing on gastric wash [39]. To this study six patients with gastric cancer and five subjects with superficial gastritis (form Beijing, China) were enrolled. The level of *Neisseria*, *Alloprevotella*, *Aggregatibacter*, *Porphyromonoas endodontalis*, and *Streptococcus mitis* was increased whereas the amount of *Sphingobium yanoikuyae* was depleted in gastric cancer. Notably, *S. yanoikuyae* is able to degrade carcinogenic compounds, thus the reduction level of this bacterium may promote carcinogenesis [39].

Interestingly, Oluwabukola Coker et al. investigated the gastric mucosal microbiome imbalance across the stages of GC [9]. This study included 81 cases regarding superficial gastritis (SG), atrophic gastritis (AG), intestinal metaplasia (IM), and gastric cancer from China. Gastric mucosal samples were analysed using 16S rRNA sequence. It was revealed that *Parvimonas micra*, *Dialister pneumosintes*, *Slackia exigua*, *Peptostreptococcus stomatis*, *Prevotella intermedia*, *Fusobacterium nucleatum*, *Prevotellaoris*, and *Catonella morbi* were significantly enriched in gastric cancer in comparison to precancerous stages [9]. The authors noted that oral bacteria were significantly more abundant in gastric cancer compared to the benign stages. Oral microbes, such as *P. stomatis*, *S. exigua*, *P. micra*, *Streptococcus anginosus*, and *D. pneumosintes,* might be involved in gastric carcinogenesis and they promote the progression of GC [9].

In systematic review regarding thirteen original articles, it was noted that gastric carcinogenesis may be linked to the increased level of several bacteria, such as *Lactobacillus coleohominis*, *Klebsiella pneumoniae*, *Acinetobacter baumannii* and decreased amount of other microbes, *e.g., Porphyromonas* spp, *Neisseria* spp., *Prevotella pallens*, and *Streptococcus sinensis* [40]. In addition, the authors emphasized that it remains unclear whether dysbiotic gut microbiota alterations are the cause or consequence of carcinogenesis [40].

Innate immunity is considered an important part of the homeostatic system keeping the balance between microbiota and host’s body. It has its role in fighting off the bacterial infection also. In inflammatory conditions the crosstalk between innate and adaptive immunity is even more evident than at times of homeostatic balance, even though the engagement of T cells in anti-bacterial response is quite late, it is considered indispensable [21, 22]. The innate cells involved in the immunological response are not only aforementioned ILC2s, but also macrophages, natural killers (NKs) and dendritic cells (DCs) residing in the lamina propria of the gastric mucosa and answering to the presence of foreign PAMPs (pathogen associated molecular patterns) by recognising them with TLRs (tool-like receptors) [41]. Adaptive immunity is at first based on the production of IgAs by specific plasma cells stimulated by ILC2s, later on it will engage T cells also [22] T cells will enhance the response of innate immune system (e.g., neutrophils and the production of antimicrobial peptides) and stimulate B cells to further produce antibodies—this time mainly IgGs [41, 42].

Whereas the outcome of the inflammatory reaction in most of the cases means a complete recovery, some specific bacteria-dependent and host-dependent factors increase the risk for cancer, e.g., GC development. *H. pylori* can acquire some specific genetic properties, increasing the risk for more intense inflammatory response mediated by IL-22 (CagA variants), IL-8 (Cag A and VacA variants), increasing neutrophils infiltration, gastric mucosal atrophy (VacA variants) and increasing bacterial adherence and colonization mediated by outer membrane proteins, i.e., BabA, HopH, and others [41, 43–45]. Host-specific features increasing the risk for GC development are linked to both innate and adaptive immunity [43]. TLRs are recognising specific bacterial antigens—PAMPs. Normally they can stimulate quite efficient reaction eliminating pathogen(s) from the body. Specific polymorphisms of TLRs can lead to a quite potent inflammatory response being a gateway to the chronic inflammation and carcinogenesis [43, 44]. The authors are not going to describe them in here as there are many papers dedicated to the topic [43, 46]. In addition, what is worth to mention, SNPs seem to be strongly ethnic-related [43, 46]. The importance of TLRs in the development, as well as in the treatment of GC, can be proven by data suggesting that TLRs can be promising targets in immunotherapy of gastric cancer [46, 47]. Apart from the TLRs polymorphisms, other elements of innate immunity can increase the risk of GC, such as specific SNPs of*CD14* gene which participate in the immune response together with TLR-4 [46, 48] and specific variants of NODs [46]. All of them, together with numerous identified SNPs in genes encoding cytokines, such as, e.g., IL-1, IL-8, IL-10, TNF-α [46], can affect the effectiveness of bacteria eradication from the body, the risk of chronic inflammation and in unfavourable outcome cancer development.

Overall, it should be emphasized that *H. pylori* is established as a main bacterium which lead to development of gastric cancer. Notwithstanding, the dysbiotic alterations of gut microbiota in GC patients seem to be more complicated and according to aforementioned data several genera (*Lactobacillus*, *Lachnospiraceae*, *Escherichia-Shigella*, *Nitrospirae*, and *Burkholderia fungorum*) are increased in these patients. In addition, some studies have also shown other changes of gastric microbiota. The difference among observed results may be associated with the methods by which the bacteria were detected. Currently, 16S rRNA sequencing is the most recommended method. In addition, gut microbiota depends on many factors and microbial biomarkers which allow to distinguish GC patients from healthy control is also strongly related to among others ethnicity.

### Fungi

Mycobiota (fungal microbiota), especially in oncological aspects, is relatively poorly investigated [49]. Nevertheless, the results of some studies have shown that fungal dysbiosis is associated with oral, pancreatic and colorectal carcinogenesis [23]. Recently, in 2021 Zhong et al. characterized the fungal microbiome in GC patients (*n* = 45) who were admitted at the First Affiliated Hospital of China Medical University, Shenyang, China [50]. It was shown that the abundances of 15 fungal biomarkers allow to distinguish patients with GC from control subjects. Notably, *Candida* (*p* = 0.000246) and *Alternaria* (*p* = 0.00341) were increased whereas the amount of *Saitozyma* (*p* = 0.002324) and *Thermomyces* (*p* = 0.009158) was decreased in patients with GC. In addition, it was also noted that *Candida albicans* was significantly elevated in GC patients and it can be assessed as a potential microbial biomarker for these patients [50]. It should be emphasized that this yeast promote carcinogenesis mainly through triggering inflammation [23].

*Candida albicans* triggering the secretion of IL-7 by subepithelial macrophages in mice gut [29] leads to the release of IL-22 by ILCs. Considering that IL-22 is associated with cancer development [51] through the impact on the tumour progression and invasion [52] and its’ level is increased in many cancers [53, 54], this ability of *C. albicans* cannot be ignored in analysing the potential impact of (dysbiotic) microbiota on the development of gastric cancer.

### Viruses

The human virome regards all viruses which are present in human body and besides bacteria and fungi, is a part of microbiota. The composition of virome depends on age, life style, and the presence of other component of microbiota [55]. Viruses can interact with other microbes, mainly bacteria. Several viruses, such as hepatitis B virus (HBV), hepatitis C virus (HCV), cytomegalovirus (CMV), human herpesvirus 8 (HHV-8), human papilloma virus (HPV), and Epstein–Barr virus (EBV), are associated with cancers [56]. For instance, HCV and HBV are involved in development of hepatocellular carcinoma [57].

It is estimated that approximately 10% of gastric carcinomas is Epstein–Barr virus associated gastric cancer (EBVaGC) [58]. Tumors which are positive for EBV present recurrent PIK3CA (80%), ARID1A (55%) and BCOR (23%) mutations as well as JAK2 amplification (25%). Moreover, around 50% of EBVaGC present amplification of 9p24.1 locus *CD274*(*PD-L1*) and *PDCD1LG2*(*PD-L2*) [3]. It is interesting to note that EBVaGC is not considered a risk factor for *H. pylori* induced GC as well as *H. pylori* infection is not considered a risk factor for EBVaGC, suggesting the involvement of different carcinogenic pathways in those instances [59].

**Fig. 1:**
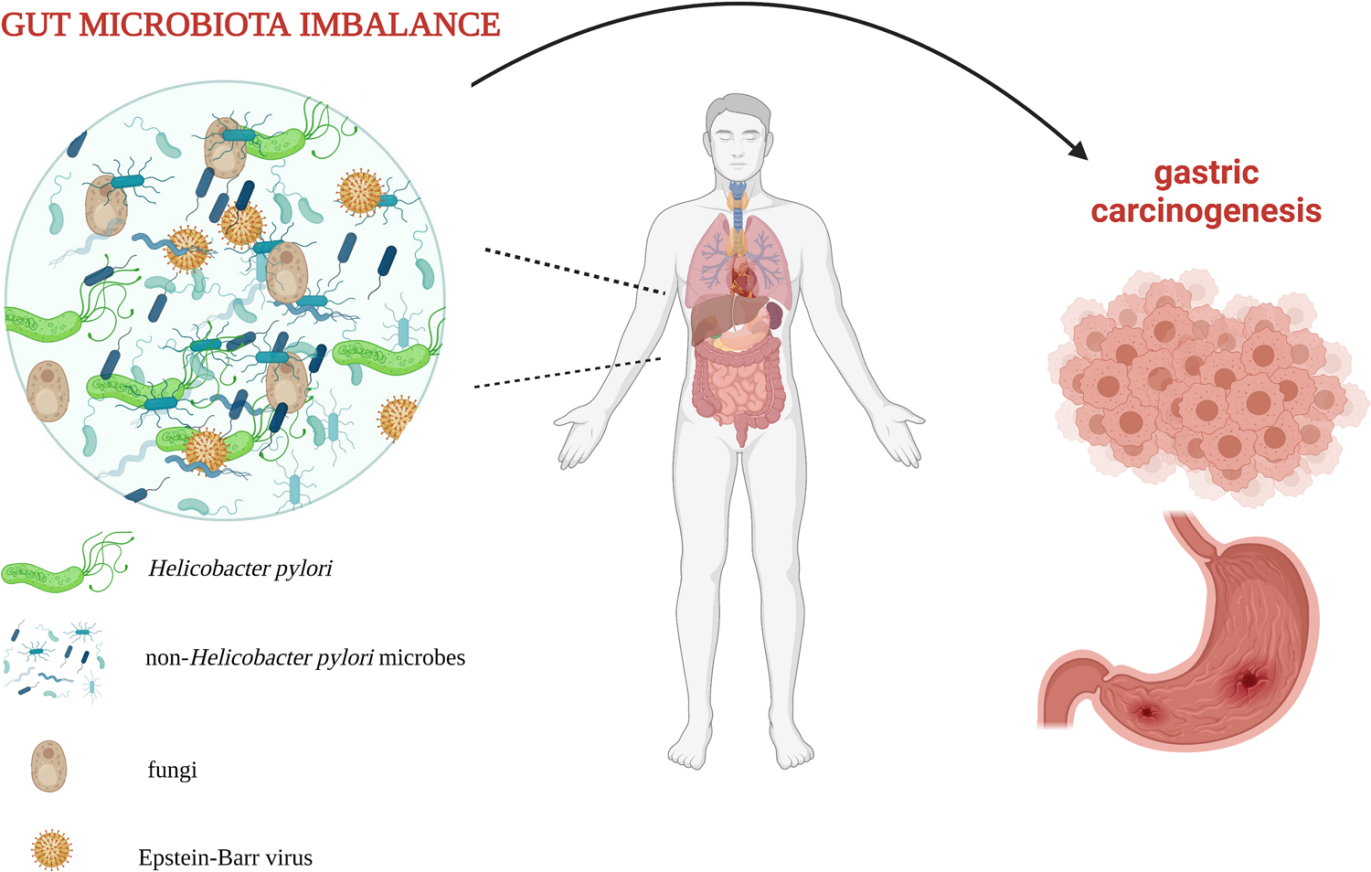
Gut microbiota imbalance and gastric carcinogenesis. Own elaboration based on literature [50, 58, 63]

As of right now, it is also well established that EBV can inhibit the proliferation of CD8^+^ T cells and lower the cytotoxicity of NK cells. That way it can establish latency and protection from the immune system actions at first and propagate more easily later. It may lead to the development of acute and chronic gastritis after some time and later it will increase the risk for tumorigenesis [41, 60]. Moreover, specific EBV miRNAs can affect the proliferation and/or apoptosis of virus-infected cells increasing the risk for the malignancies formation [60, 61]. In general, the pathways involved in, e.g., cytokines activity, immune response, leukocytes migration are deregulated and will impact the risk of GC development in EBVaGC carriers [62] (Fig. 1).

## The association between gut microbial metabolites and gastric cancer

In the last few years, the involvement of pathogenic and commensal bacteria on the pathogenesis of cancer has been confirmed. Bacteria can affect several aspects of cancer, such as prevention, induction, response to treatment and development of resistance. These effects can be caused not only by bacterial genotoxins (e.g., colibactin, CagA, VirA, P37, IpgD) but also by common product of bacterial catabolism. Several data shown the effect of gut microbial metabolites including shot-chain fatty acids (SCFAs), polyamines, and product of polyphenol and tryptophan catabolism, on cancer development and progression. Bacterial metabolites can trigger alterations in the cell cycle and regulate immune response through transcriptional and epigenetic metabolites, playing a crucial role in carcinogenesis. However, the mechanism of these effect is less understood yet and further studies of the relationship between bacterial metabolites and cancer are needed.

### Short-chain fatty acids (SCFAs)

SCFAs, including butyrate, propionate, and acetate, are produced by gut microbiota from fermentable non-digestible carbohydrates [64, 65]. Acetate and propionate are mainly produced by *Bacteroidetes phylum*, whereas butyrate by *Firmicutes* [64]. Diet, age and conditions/diseases can alter their concentration and proportion [63]. SCAFs can active various cellular mechanisms related mainly to the prevention of carcinogenesis. This impact is associated with the regulation of cellular pathway (*e.g*., Akt/mTOR and MEK/ERK signalling pathways), transcription factor (downregulation of NF-kB, and epigenetic regulation (e.g., inhibition of HDACs–histone deacytylases–activity, DNA methylation, histone phosphorylation and methylation), resulting in regulation of the cell cycle, apoptosis and regulation of immune response [66, 67]. Multiple studies have confirmed that butyrate plays a significant role in human body and represent the only SCFA in which the anticarcinogenic activity is known [66, 68]. The main butyrate-producer is *Faecalibacterium prausnitzii* belonging to the next-generation probiotics group [64]. It is a source of energy for colonocytes, enhances gastrointestinal immunity and maintains intestinal barrier integrity [69, 70]. However, butyrate, may also promote carcinogenesis via increase of aberrant epithelial cells proliferation [58, 71].

Matthews et al. investigated the impact of two SCFAs, i.e., propionate and butyrate on cell viability as well as cell cycle regulation in a human gastric cancer cell line (Kato III) [72]. The cells lines were incubated with SCFAs for 24, 48, and 72 h. Induction of apoptosis and changes of cell cycle were assessed using flow cytometry. SCFAs induced apoptosis and necrosis in Kato III cells. It was also noted that the effect obtained after using butyrate was significantly greater compared to the propionate. Interestingly, sodium butyrate is able to inhibit cell proliferation and induce differentiation in a variety of cancer cells [73]. It implies alterations in the proliferation of apoptosis-related genes in human gastric cells line, decreasing the expression of FAK (focal adhesion kinase) and increases the expression of DAPK1/2 which induces apoptosis [74]. Treatment with sodium butyrate leads to the acetylation of p53 that induces p21 (CDKN1A), which inhibits the activity of cyclin-dependent kinase 2 (CDK2) in G1/S phase with the cycle arrest in G1 [75]. These results indicate that the anticancer effect of SCFAs could enhance the efficacy of chemotherapeutics used to treat gastric cancer [72].

### Polyamines (PAs)

Polyamines (PAs) including putrescine, cadaverine, spermidine and spermine, are microbial metabolites synthesized in the gut mainly by *Firmicutes sp*. [76, 77]. Polyamines functions are associated with maintaining cell wall stability, synthesis of siderophores, protection against free radicals and acids [78]. As shown in cell culture and animal models studies, altered levels of intracellular PAs and change in their metabolism are associated with several types of cancers [79]. Ornithine decarboxylase (ODC) and adenosylmethionine decarboxylase 1 (AMD1) are key enzymes involved in biosynthesis of polyamines. Increased levels of ODC activity and, therefore, increased PAs concentration has been associated with colorectal cancer development [80]; conversely, a diet enrich of probiotics (*Bifidobacterium sp, Lactobacillus sp. and Streptococcus sp*.) in murine model, resulted in a decrease of PAs concentration with anticancer effects [81]. The potential function of AMD1 in human gastric cancers is unknown. Recently Xu et al. shown that knock down of AMD1 in a tumor xenograft model, suppressed the tumor growth in vivo and that the inhibition of AMD1 by an inhibitor SAM486A in human gastric cancer cells arrested cell cycle progression during G1-to-S transition [82]. Moreover, in this study, patients with high expression of AMD1 had a much shorter overall survival than those with normal/low expression of AMD1. These results confirmed the tumorigenic effect of AMD1 on human gastric cancers and its impact on the prognosis of the patients [82].

### N-nitroso compounds (NOCs) and lactate

It is well-established that nitrosating agents play an important role in gastric carcinogenesis [83]. N-nitroso compounds (NOCs) derive in part from diet (e.g., processed meat, smoked fish, and certain vegetables) as well as from endogenous synthesis [84]. Several bacteria, such as *Veillonella, Clostridium, Haemophilus, Staphylococcus, Neisseria, Lactobacillus,* and *Nitrospirae,* contribute to gastric carcinogenesis by stimulating the production of NOCs [85]. Epidemiologic studies have shown that patients with GC have higher NOC levels than healthy subjects. Nitrosating or nitrate—reducing bacteria were found to be more abundant in GC patients than control subjects, although the difference between the two groups was not statistically significant [86]. Higher nitrate and nitrite reductase activities associated with the microbiome were observed in GC rather than in chronic gastritis [37].

In vitro and in vivo experiments have demonstrated that lactic acid bacteria stimulate the generation of ROS that cause DNA damage and enhance the formation of NOCs that induce mutagenesis, angiogenesis, and protooncogene expression and inhibit apoptosis [87].

The abundance of lactic acid bacteria was shown to be increased in patients with GC. Lactate, metabolized by lactic acid bacteria, is a source of energy for cancer cells [88] and plays a regulatory role in various aspects of carcinogenesis including tumor angiogenesis, immune escape, tumor cell migration, and metastasis [89]. Higher levels of L- and D-lactate and lactate dehydrogenase were recorded in patients with gastric cancer than in those with gastric ulcers and healthy controls [90].

### Others bacterial metabolites

Other metabolites, such as polyphenols and tryptophan, can participate in the carcinogenesis. Most of the polyphenol metabolites are produced by gut bacteria of *Clostridium sp.* and *Eubacterium sp*. and by probiotic bacteria (*e.g., Bifidobacterium, Lactobacillus*) [91, 92]. Their anticarcinogenic effect is due to the impact on the cell cycle, the apoptosis induction and to the inhibition of proinflammatory cytokines synthesis [93]. Another metabolite, i.e., tryptophan is mainly metabolized by *Firmicutes* (*Clostridium sporogenes, Ruminococcus gnavus* and *Lactobacillus sp*.) but also by some opportunistic pathogenic bacteria [94, 95]. More evidence indicate that Trp metabolism have an essential role in suppression of anticancer immune responses and in an increase in malignant properties of cancer cells, leading tumor progression [96, 97]. However, although the effect of these metabolites on carcinogenesis is now evident, available data on the role of bacteria in this process are very little yet. Further studies may provide new understanding of the relationship between diet, gut microbiota and carcinogenesis to improve both anticancer therapy and cancer prevention especially in GC patients [98].

## Gastric cancer and gut microbiota-related studies which are currently ongoing worldwide

Currently, several studies regarding gastric cancer and gut microbiota-related aspects are registered in *ClinicalTrials.gov* system (terms: gastric cancer, gut microbiota; accessed on 7 August 2021; 12 trials: NCT04980950, NCT04660058, NCT04638959, NCT04365946, NCT04198051, NCT04022109, NCT04889859, NCT02833363, NCT03250091, NCT02332213, NCT04015466, NCT03228095). These trials are presented in Table 1.

**Table 1:** The selected projects registered in ClinicalTrials.gov system which regard the link between gastric cancer and gut microbiota-related aspects

ClinicalTrials.gov identifier	Title of project	Participants (*n*)	Conditions/diseases	Current status	Treatment/interventions	Researcher/responsible party
NCT04980950	"The Impact of Immunonutrition on Gut Microbiota-related Aspects in Colorectal Cancer and Gastric Cancer Patients"	80	Gastric cancer Colorectal cancer	Not yet recruiting	Administration of dietary supplement: 1.Intervention group Impact Oral Nestlé Health Science and Cubitan^®^ Nutricia 2.Control group Nutridrink^®^ Nutricia and Resource 2.0 Nestlé Health Science	Karolina Kaźmierczak-Siedlecka, Medical University of Gdansk
NCT04660058	“Associations Among Serum and Gastric Juice Bile Acid Profile, Bile Acid-microbiota Cross-talk in Stomach and the Development of Gastric Cancer”	80	Bile Reflux Gastric Cancer Precancerous Lesion	Enrolling by invitation	The aim is to explore the associations among bile acid profile, bile acid-microbiota cross-talk, and the development of gastric cancer	Yongquan Shi, Xijing Hospital of Digestive Diseases
NCT04638959	“Study of Faecal Bacteria Detection in Early Screening and Diagnosis of GC”	110	Gastric cancer Chronic gastritis	Recruiting	Collecting of gastric tissue and stool samples	Jing-yuan Fang, Shanghai Jiao Tong University School of Medicine
NCT04365946	“Microbiome Analysis in Gastric Intestinal Metaplasia and in Gastric Cancer and Subtypes Correlation”	80	Gastric intestinal metaplasia Gastric cancer	Active, not recruiting	Gastroscopy	George Pappas-Gogos, University Hospital, Ioannina
NCT04198051	“The Clinical Study of Adjuvant Chemotherapy on Intestinal and Urethral Flora in Patients With Gastric and Colon Cancer”	120	Gastric cancer Colon cancer	Recruiting	The use of adjuvant chemotherapy	Xiaonan Cui, The First Affiliated Hospital of Dalian Medical University

## Conclusions

In conclusion, considering potential bacterial, fungal and viral pathomechanism as a sole agent leading to tumorigenesis and gastric cancer development is very difficult. *H. pylori* infection is considered the most important/the most researched causative agent for GC development, but in reality, it is a matter of coexistence and net-effect of many influences at the time. The association between gut microbial metabolites and GC is also observed especially in case of SCFAs, PAs, NOCs, and lactate. Therefore, it should be emphasized that the modulation of gut microbiota should regard not only its composition but also activity aspects.
